# A Survey of Obstetric Anaesthesia Services and Practices in the United Kingdom

**DOI:** 10.7759/cureus.70851

**Published:** 2024-10-04

**Authors:** James O'Carroll, Liana Zucco, Eleanor Warwick, Gill Arbane, Ramani Moonesinghe, Kariem El-Boghdadly, Nan Guo, Brendan Carvalho, Pervez Sultan

**Affiliations:** 1 Targeted Intervention, University College London, London, GBR; 2 Anaesthesia and Perioperative Medicine, Guy’s and St Thomas’ NHS Foundation Trust, London, GBR; 3 Anesthesiology, Perioperative and Pain Medicine/Obstetrics, Stanford University School of Medicine, Stanford, USA

**Keywords:** enhanced recovery after caesarean, national health service, obstetric guidelines, quality of care, quality of recovery, survey

## Abstract

Background

Variability in obstetric anaesthetia practice and care delivered within the UK is under-explored. The ObsQoR study explored structures, processes, and outcomes of obstetric anaesthesia in 107 hospitals within the UK’s National Health Service, and the results of the hospital-level survey are reported here.

Methods

Hospitals were surveyed to assess obstetric anaesthesia provision, practice, and care delivery. Questions explored staffing, service provision and training, facilities present, clinical practices, outcome measurement, and key indicators of quality in obstetric anaesthesia.

Results

We received responses from 106 participating hospitals, representing 69% of all UK obstetric units. One hundred (94%) hospitals had a dedicated consultant obstetric anaesthetist within working hours, with 27 (25%) of hospitals’ duty anaesthetists having additional clinical responsibilities outside the care of obstetric patients outside of working hours. Around 102 hospitals (98%) offer multidisciplinary team training, of which 95 (93%) use a simulation-based method. Dedicated high-risk antenatal clinics were present in 50 (47%) hospitals. The majority of hospitals provide written patient information in multiple languages for discussing obstetric anaesthesia options (77, 82%). Seventy-three hospitals (69%) use point-of-care testing to estimate haemoglobin concentration. Labour epidural analgesia is most commonly delivered via patient-controlled epidural analgesia in 80 (76%) hospitals, and the incidence of post-dural puncture headaches was recorded by 80 (76%) hospitals.

Conclusions

These results demonstrate variation in the provision of staffing, facilities, clinical practices, and outcome measurements across the UK. To deliver safe and equitable care across the UK, there needs to be standardisation of anaesthetic peripartum care based on national recommendations and the benchmarking and measurement of appropriate markers of quality.

## Introduction

There are approximately 680,000 births each year in the United Kingdom [1], and obstetric operative procedures constitute a large proportion of elective and emergency surgical cases performed in the National Health Service (NHS) [2,3]. Obstetric anaesthetists are an integral part of the team involved in peripartum care, in addition to providing labour analgesia or anaesthetic intervention in an estimated 65% of those around the time of delivery [4].

The provision of optimal hospital care for labour and delivery is essential to reduce preventable maternal morbidity and mortality. The most recent Mothers and Babies: Reducing Risk through Audit and Confidential Enquiries across the UK (MBRRACE-UK) report concluded that deaths attributed directly to anaesthesia are very rare, but improvements in the overall peripartum care delivered to 38% of those that died between 2018 and 2020 may have led to a difference to the outcome, with only 22% receiving good quality care [5]. It is known there is variability in obstetric anaesthesia practices within the US and Europe [6-8]. Less is known regarding the variability in the UK and its impact on outcomes. Furthermore, contemporary national data regarding obstetric analgesia or anaesthetic interventions and postpartum recovery are lacking [9-11].

There have been best practice recommendations outlined for the provision of obstetric anaesthesia and the delivery of care during the peripartum period. These guidelines include recommendations regarding appropriate staffing, service provision and training, care facilities and equipment, clinical practices, and outcome measurement [7,12-22]. Measuring adherence to these guidelines, comparing performance to established standards of practice, and evaluating available facilities can be used to benchmark current practice, compare the performance of different hospitals, and help identify key improvement priorities.

The ‘Quality of Recovery in Obstetric Anaesthesia, a Multicentre Study’ (ObsQoR) was a prospective study conducted in UK NHS obstetric units in October and November 2021, which aimed to evaluate postpartum recovery following anaesthetic interventions across England, Scotland, Wales, and Northern Ireland [23]. As part of this study, an institutional survey was planned and sent to each participating study lead to evaluate site-specific factors related to the institution’s provision of anaesthetic peripartum care and alterations related to COVID-19. The institutional survey's purpose was to investigate hospital-level variations in staffing, facilities, and clinical practices that may be associated with the quality of peripartum care and postpartum recovery.

## Materials and methods

We conducted an institutional-level survey as part of the ObsQoR study in the UK. This was a collaboration between University College London Hospital, London, Guy’s and St Thomas’ Hospital, London, UK, and Stanford University, California, USA. All NHS obstetric units with anaesthetic services were invited to participate in the ObsQoR study via the National Institute for Health Research clinical research networks and trainee anaesthetic networks. Prospective ethical approval was obtained for the study (South Central Berkshire B REC).

The ObsQoR study aimed to assess inpatient and outpatient postpartum recovery following anaesthetic or analgesic interventions, including evaluation of demographic, obstetric, anaesthetic, and institutional factors that may impact the quality of recovery. To further examine hospital-level factors of structures, processes, and outcome measures that may affect the quality of postpartum recovery, an institutional survey was designed. This included questions relating to staffing, service provision and training, facilities present, clinical practices, outcomes collected locally, and key quality and safety performance indicators in obstetric anaesthesia. One hundred and seven sites participated in the ObsQoR study, representing 69% of the 156 UK obstetric units.

The institutional survey was developed using a consensus method to evaluate standards of obstetric anaesthesia care, which may affect the quality of postpartum recovery. The core study group (JOC, LZ, EW, KE, BC, and PS) convened to design survey questions mapped against guidelines, currently identified best practices and expert opinion. Draft questions were circulated amongst core study group members for feedback and evaluation, modified over three rounds, and then piloted in six selected participating obstetric units (Appendix 1). The final survey was distributed via email to all 107 participating ObsQoR sites at the start of a two-week patient enrolment and recruitment period.

Institutional surveys were completed by local ObsQoR principal investigators, with assistance from clinical leads for obstetrics, anaesthesia, and midwifery, where needed. Results were entered onto a web-based platform (FormAssembly; www.formassembly.com; Veer West LLC, Bloomington, IN, USA). Reminder emails were sent to all ObsQoR sites weekly to encourage a high response rate, prompting survey completion until study closure in November 2021. Data were reviewed centrally, and errors or missing data were verified and clarified with local study teams.

Statistical analyses were performed using Stata (v.14.0) (StataCorp LLC, College Station, TX, US) and Microsoft Excel (v.16.5) (Microsoft Corp., Redmond, WA, US). Descriptive statistics for normally distributed continuous data are reported as mean (SD), and non-normally distributed data are reported as median (IQR; range), with comparisons between hospital sizes made using ANOVA and Kruskal-Wallis tests, respectively. Categorical data are presented as counts (percentage) and compared using the chi-square test of Fisher's exact test where appropriate. A p<0.05 was considered significant, and all tests were two-tailed. Missing data or incomplete items were not included in the analyses. Additional free-text responses were examined for trends, categorised, and aggregated using thematic analysis by two authors (JO and LZ).

## Results

**Figure 1:**
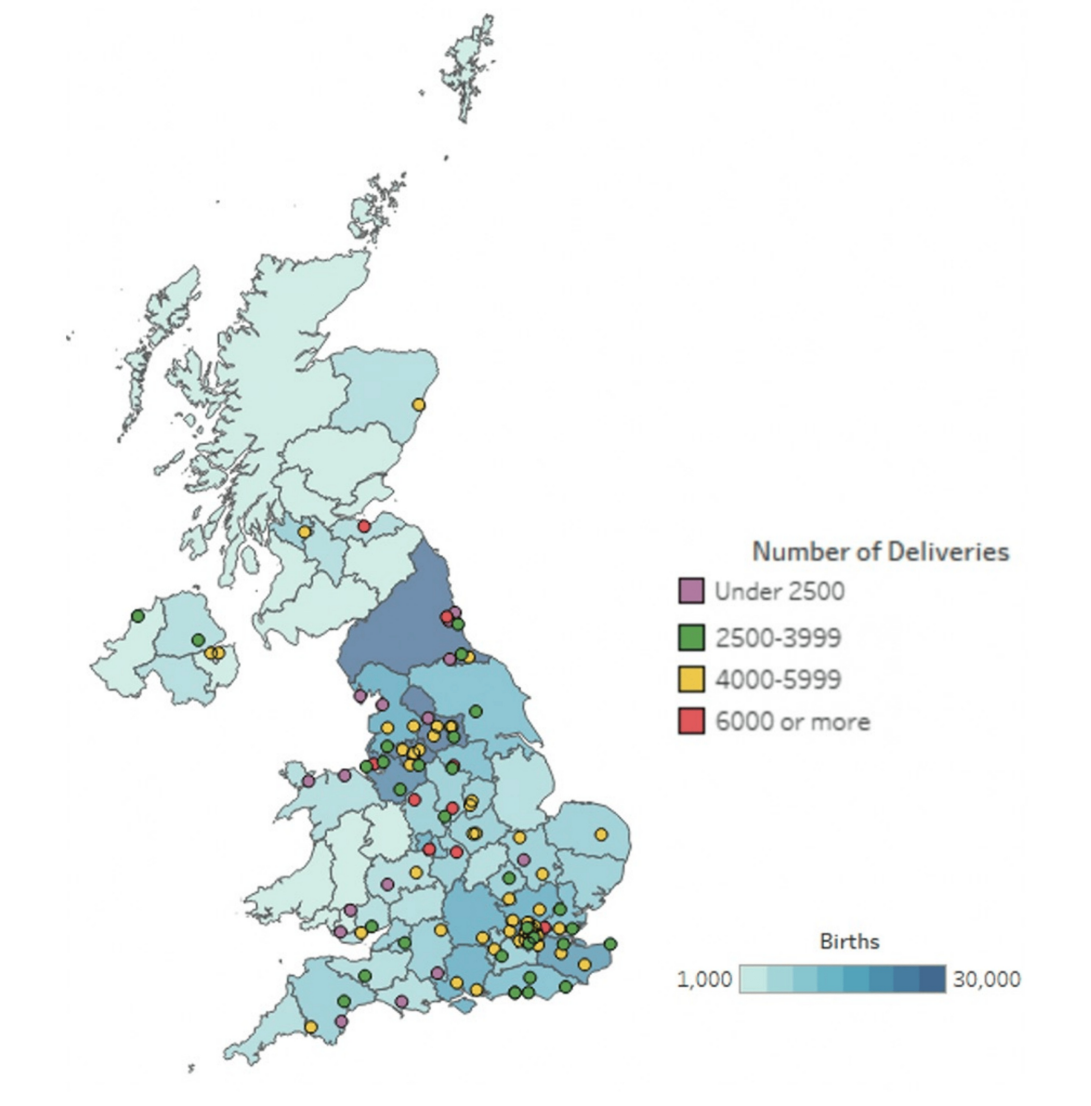
Map of the United Kingdom with participating hospitals shown, their annual number of deliveries, and the total number of births in that region in 2020. [23] Usage permission obtained (RightsLink Printable License)

**Figure 2:**
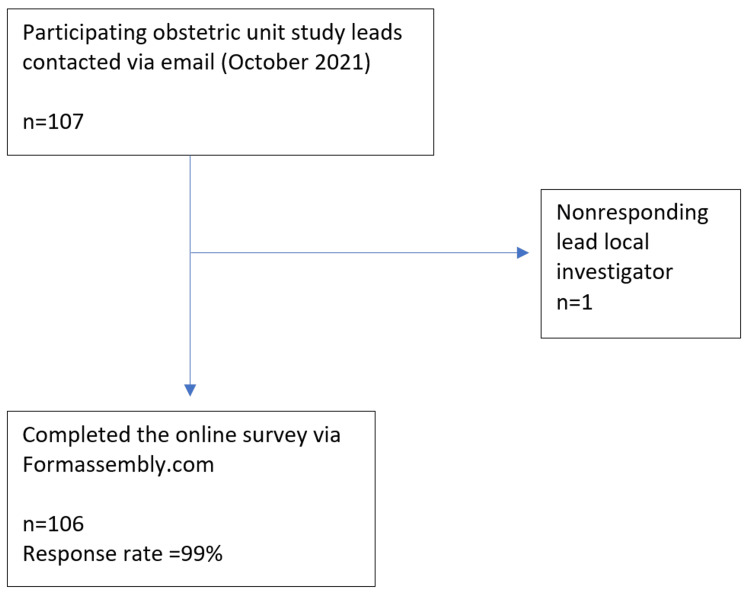
Participant flow diagram.

Survey results were received from 106 out of 107 participating ObsQoR hospital sites, with a response rate of 99%. This represents 69% of the 156 obstetric units in the UK (excluding the Crown dependencies). These hospitals were from 78 English NHS Trusts, three Scottish NHS Boards, four Welsh Health Boards, and four Northern Irish Health and Social Care Trusts. The number of deliveries reported by region and participating obstetric units is presented in Figure 1, and the participant flow diagram is provided in Figure 2. The reported median (IQR; range) annual number of deliveries from the previous year in the included institutions was 4350 3000 - 5366, 1000 - 8200. The results are presented for obstetric units in the UK relating to staffing, service provision and training, facilities present, clinical practice, follow-up, quality and safety indicators, and outcome measurement.

Staffing, service provision, and training 

One hundred hospitals (94%) have a dedicated consultant-level anaesthetist present in the labour ward during working hours. Eighty-one (76%) reported having an additional dedicated consultant-level anaesthetist for elective obstetric theatres. Out of hours, a consultant-level anaesthetist dedicated to obstetrics alone was reported by 23 hospitals (22%). All reported at least one duty anaesthetist out-of-hours, with 27 hospitals (25%) stating this anaesthetist has commitments in other clinical areas of the hospital outside of the labour and delivery suite. This was most commonly reported by hospitals with less than 2500 deliveries per year. The majority of hospitals provide multidisciplinary team (MDT) training (102/104; 98%), and this is mostly simulation-based. Results for staffing provision and training, including a breakdown according to the number of deliveries performed by each institution per year, are summarised in Table 1.

**Table 1:** Staffing, service provision, and training at each included hospital. ED: emergency department; CSE: combined spinal-epidural; ODP: operating department practitioner Values are numbers (proportion).

Variable	n (%)	Number of deliveries per year				
		<2500	2500-3999	4000-5999	>6000	p
Obstetric staffing – Consultant anaesthetist						
Dedicated anaesthetic consultant for labour ward during working hours	100/106 (94%)	14/16 (88%)	28/30 (93%)	45/46 (98%)	13/14 (93%)	0.288
Time anaesthetic consultant present for labour ward (n=100)						
Working hours or until 18:00	75/100 (75%)	12/14 (86%)	26/28 (93%)	33/45 (73%)	4/13 (31%)	<0.001
Present until between 18:00- 21:00	22/100 (22%)	2/14 (14%)	1/28 (3.5%)	11/45 (24%)	8/13 (62%)	0.001
Present after 21:00	3/100 (3%)	0/14 (0%)	1/28 (3.5%)	1/45 (2%)	1/13 (7.7%)	0.001
Dedicated anaesthetic consultant for elective obstetric theatres (i.e. not expected to cover obstetric emergency work)	81/106 (76%)	8/16 (50%)	17/30 (57%)	43/46 (92%)	13/14 (93%)	<0.001
Dedicated obstetric anaesthetic consultant on-call out of hours (i.e. not cross-covering other specialities)	23/106 (22%)	1/16 (6.3%)	1/30 (3.3%)	13/46 (28%)	8/14 (57%)	<0.001
Obstetric staffing – Non-consultant anaesthetist						
Non-consultant grade on-call anaesthetist with commitments to other clinical areas during working hours (e.g. general theatres, ED, critical care; n= 105)	13/105 (12%)	6/16 (38%)	3/30 (10%)	4/45 (8.8%)	0/14 (0%)	0.017
Non-consultant grade on-call anaesthetist with commitments to other clinical areas out of working hours (e.g. general theatres, ED, critical care)	27/106 (25%)	10/16 (63%)	11/30 (37%)	6/46 (13%)	0/14 (0%)	<0.001
Multi-disciplinary staff & training						
Anaesthetic assistants/ODPs routinely attend labour rooms to assist with labour analgesia	39/105 (37%)	5/16 (31%)	6/29 (21%)	20/46 (44%)	8/14 (57%)	0.079
Dedicated theatre team and obstetric staff to cover elective caesarean lists	83/106 (78%)	9/16 (56%)	21/30 (70%)	39/46 (85%)	14/14 (100%)	0.010
Presence of obstetric medicine clinician within the department	40/104 (38%)	4/15 (26%)	7/29 (24%)	18/46 (39%)	11/14 (79%)	0.006
Multidisciplinary training is provided	102/104 (98%)	16/16 (100%)	29/30 (97%)	44/45 (94%)	13/14 (93%)	0.437
Multidisciplinary training is simulation-based	95/102 (93%)	16/16 (100%)	28/29 (97%)	40/44 (91%)	11/14 (85%)	0.334

Available facilities present at each obstetric unit

The facilities available at each obstetric unit site related to antenatal clinics, the provision of patient information, escalation pathways, and access to emergency equipment and results are summarised in Table 2. Ninety of 106 units (85%) offer a dedicated anaesthetic antenatal clinic, with 50/103 (49%) offering a clinic specifically for high-risk parturients. Trust-approved information about analgesia and anaesthesia for labour and delivery is given to all as part of routine antenatal care in 81 (77%) hospitals. Where written information is given, it is available in multiple languages in 80% (77/96). Where this is not available, interpreter services can be accessed in 94% (17/18) of hospitals. Obstetric medicine physicians are present in 40 (38%) hospitals, and 49 (47%) have a lead clinician for critically ill patients, with 80 (76%) having clear guidelines for the escalation to critical care. Only 44 (41.9%) hospitals have a dedicated obstetric high-dependency unit (HDU). The level of care delivered on the obstetric HDU is level 1 (defined as being suitable for patients at risk of deterioration in 24 (55%), “enhanced maternal care” in five (11%) hospitals, and level 2 (suitable for patients on single organ support) in 15 (34%) of hospitals.

**Table 2:** Summary of the available facilities present at each hospital. BMI: body mass index; Hb: haemoglobin; MDT: multidisciplinary team (n=number of hospitals that responded to the question, if not stated n=106)

Dedicated obstetric facilities & high dependency:						
Number of dedicated obstetric theatres per hospital	n (%)	<2500 n= 16	2500-3999 n=30	4000-5999 n=46	>6000 n=14	p
1	30/106 (28%)	10 (62.5%)	14 (47%)	6 (13%)	0 (0%)	<0.001
2	69/106 (65%)	6(37.5%)	14 (47%)	38 (83%)	11 (79%)	
3	7/106 (6.6%)	0 (0%)	2(6.7%)	2 (4.3%)	3 (21%)	
	n (%)					
Dedicated obstetric recovery in working hours	91/106 (86%)	12/16 (75%)	25/30 (83%)	40/46 (87%)	14/14 (100%)	0.238
Dedicated obstetric recovery out of working hours	76/106 (72%)	10/16 (63%)	23/30 (77%)	32/46 (70%)	11/14 (79%)	0.715
	n (%)					
Dedicated obstetric high-dependency unit	44/105 (42%)	2/14 (13%)	8/29 (28%)	23/46 (50%)	11/14 (88%)	0.001
Level of care within obstetric high dependency unit	n (%)					0.293
Level 1	24/44 (55%)	2/2 (100%)	6/8 (75%)	13/23 (57%)	3/11 (27%)	
Enhanced maternal care	5/44 (11%)	0/2 (0%)	0/8 (0%)	2/23 (8.7%)	3/11 (27%)	
Level 2	15/44 (34%)	0/2 (0%)	2/8 (25%)	8/23 (35%)	5/11 (45%)	
Access to emergency equipment/treatments	n “yes” (%)					
Difficult airway equipment immediately available	105 /105 (100%)	16/16 (100%)	29/29 (100%)	46/46 (100%)	14/14 (100%)	
O rhesus negative packed red cells, within 5 minutes, at all times of day	102/106 (96%)	15/16 (94%)	28/30 (93%)	45/46 (98%)	14/14 (100%)	0.630
Rapid fluid infuser device	97/105 (92%)	14/16 (88%)	27/29 (93%)	43/46 (94%)	13/14 (93%)	0.902
Point-of-care testing devices (on the labour ward or in labour theatres)	93/106 (88%)	14/16 (88%)	27/30 (90%)	38/46 (83%)	14/14 (100%)	0.432
Blood gas analyser	90/106 (85%)	13/16 (81%)	25/30 (83%)	38/46 (83%)	14/14 (100%)	0.382
Hb analyser	73/106 (69%)	11/16 (69%)	22/30 (73%)	31/46 (67%)	9/14 (64%)	0.927
Coagulation analyser including thromboelastography	36/106 (34%)	6/16 (38%)	7/30 (23%)	14/46 (30%)	9/14 (64%)	0.055
Caesarean delivery guidelines and enhanced recovery programmes						
Guideline for the management elective caesarean deliveries	97/105 (92%)	16/16 (100%)	27 /29 (93%)	41/46 (89%)	13/14 (93%)	0.648
Enhanced recovery programme after caesarean delivery	74/106 (70%)	9/16 (56%)	19/30 (63%)	33/46 (72%)	13/14 (93%)	0.116

Clinical practices in obstetric anaesthesia

The method of administration for epidural analgesia most utilised was patient-controlled epidural analgesia (PCEA) in 80 of the 106 hospitals (75%), and 27 (26%) used a programmed intermittent epidural bolus (PIEB) technique. Fentanyl 2 µg.ml^-1^ with bupivacaine 0.1% was the most frequently reported epidural analgesia solution used in 101 (95%) hospitals. Fifty-two (49%) hospitals offer remifentanil patient-controlled analgesia (PCA) as an option for labour analgesia.

For elective caesarean delivery, diamorphine was the most commonly used intrathecal opioid (99/106; 93%), which is administered most frequently at a dose of 300 µg (85/99; 86%). Phenylephrine is most commonly administered as an infusion (99/106; 93%), with five hospitals using alternative agents to manage intraoperative hypotension (metaraminol/ephedrine bolus in three and metaraminol infusion in two). Post-caesarean delivery analgesia was standardised in the majority of hospitals following regional or general anaesthesia in 105/106 (99%) and 88/106 (83%), respectively. Intra-operative patient warming was routinely used in 79 units (74.5%) and most commonly delivered using cabinet-warmed fluids. Table 3 outlines the clinical practices relating to labour analgesia, intraoperative anaesthesia, and postoperative pain management.

**Table 3:** Clinical practices in obstetric anaesthesia. PCEA: patient-controlled epidural analgesia; PIEB: programmed intermittent epidural bolus; LA: local anaesthesia; PCA: patient-controlled analgesia (n= number of sites that responded to the question, if not stated n=106)

Variable	n (%)
Labour analgesia and anaesthesia	n (%)
Sites reporting method of delivery of epidural analgesia (multiple options possible) (n=106	
PCEA	80 (75%)
PIEB	27 (26%)
Clinician bolus	26 (25%)
Continuous infusion	25 (24%)
Intrathecal anaesthesia dosing (for elective caesarean deliveries)	
Most commonly used dose of intrathecal fentanyl, if used (n=50)	
< 15 µg	3 (6%)
15 µg	26 (52%)
>15 µg	21 (42%)
Most commonly used dose of intrathecal diamorphine, if used (n= 99)	
<300 µg	2 (2%)
300 µg	85 (86%)
>300 µg	12 (12%)
Most commonly used dose of intrathecal morphine, if used (n= 41)	
100 µg	37 (90%)
150 µg	4 (9.8%)
Remifentanil PCA available as an option for labour analgesia	52 (49%)
Intraoperative management	
Methods of intraoperative warming (n= 106)	
Routine use of forced air warmer	11 (10%)
Routine use of warmed fluid via warming cabinet	38 (36%)
Routine use of warmed fluid via fluid warmer	26 (25%)
Routine use of warmed blankets	17 (16%)
No active measures are routinely taken to manage body temperature	27 (26%)
Post-operative analgesia	
Standardised post-operative analgesic regimen for caesarean sections (n=106)	
Under regional anaesthesia	105 (99%)
Under general anaesthesia	88 (83%)

Follow-up, quality and safety indicators, and outcome measurement

Table 4 details obstetric anaesthesia follow-up practices, outcome measurement, and key indicators of quality recorded in the preceding year at each participating hospital. Routine postpartum follow-up by an anaesthetist occurs in 94 (90%) hospitals following any anaesthetic invention. Eighty hospitals (76%) track their local incidence of post-dural puncture headache (PDPH) as a quality measure, with the incidence reported as median (IQR; range) 0.96% (0.6-1.20) (0.24-2.9) and 99 (93%) have standardised guidelines to follow-up patients with PDPH. Survey responses indicated that local recording of the numbers of elective and emergency caesarean delivery rates occurs in 75% and 67% of hospitals, respectively. The median reported (IQR; range) general anaesthesia rate for caesarean delivery was 1.63% (1.24-2.13, 0.13-4.3). Achievement of adequate pain relief within 45 minutes from the placement of an epidural/combined spinal epidural (CSE) was recorded by 14 hospitals (13%), with seven of these actively auditing this metric. Table 4 details the follow-up, quality indicators, and outcome measurements of each participating hospital.

**Table 4:** Follow-up, quality and safety indicators, and outcome measurement. CSE: combined spinal epidural; n: number of hospitals reporting data

Follow-up and complications	n/n (%)	%	Median	IQR
Routine postpartum follow-up by anaesthesia team following any anaesthetic/analgesic interventions/care during admission	94/105	90		
Recording of achievement of adequate pain relief 45 minutes after the placement of an epidural/CSE	14/104	13		
Actively audit adequate pain relief 45 minutes after the placement of epidurals/CSE	7/14	50		
Percentage of patients receiving adequate analgesia is achieved in 45 minutes following epidural placement (n=7)			91.5	86-90
Recording of the incidence of post-dural puncture headache by institutions	80/106	76		
Percentage reported incidence of patients with post-dural puncture headache in the previous year			0.96	0.60-1.20
Standardised guidelines for follow-up of post-dural puncture headache or other complications	99/106	93		
Recording of delivery rates, interventions and complications for the preceding year				
Estimated number of deliveries			4350	3000-5366
Annual number of epidurals and/or labour analgesia interventions (n=81)			855	490-1266
Percentage of deliveries under general anaesthesia (n=77)			1.63	1.24-2.13

## Discussion

This survey is the first to evaluate the hospital-level variation in staffing, facilities, clinical practices, quality indicators, and outcome measurements related to obstetric anaesthesia across units in the UK. It encompasses mapped indicators relating to Donabedian’s structure, process, and outcomes model, which can be used as a framework to measure the quality of patient care during the inpatient anaesthetic peripartum period. The authors were independent of the institutions collecting the data. We show that there are differences in the obstetric anaesthesia provision across a large number of geographically disparate hospitals in the UK.

There are published recommendations on the appropriate staffing levels for obstetric units in the UK, including that consultant anaesthetists should be allocated for full-day time working during weekdays to provide urgent and emergency care [16]. Six hospitals reported not having this provision within working hours, with two having more than 4000 deliveries per year. A recently published survey concentrating on consultant obstetric anaesthesia-programmed activities found similar percentages for dedicated on-call and surgical lists for elective caesarean delivery, highlighting the need to assess non-consultant-delivered staffing levels [24]. We found disparity evident out of hours, with over a quarter of sites reporting that their obstetric anaesthetist has clinical responsibilities outside the labour ward, for example, critical care or the emergency department. The Ockenden Report, an independent review of maternity services at Shrewsbury and Telford NHS Trust investigating neonatal and maternal harm, highlighted the effect of a lack of an available, appropriately trained workforce. It gave specific recommendations for obstetric anaesthesia as well as stated staffing as a contributing factor to failures, ultimately impacting the quality and safety of care delivery [25]. The report also emphasised the importance of MDT learning and team-based training to foster a safety culture and maintain improved clinical performance. Simulation-based MDT training appears to be widely adopted across the UK, and this type of training has been shown to improve performance and clinical outcomes [26,27].

NHS hospitals should provide accessible information to people about their care and treatment so that there is informed shared decision-making. This should include information about analgesia and anaesthesia available during peripartum care. The information should be accessible and freely available. Accessibility of information would include having a translator or translated information available for people unable to understand English. Our survey found that 77% of hospitals reported that trust-approved information about analgesia and anaesthesia for labour and delivery was given to all as part of their antenatal care. It has previously been highlighted the paucity of adequate information given to patients during pregnancy, with almost half of patients not recalling they received any information regarding anaesthesia for caesarean delivery [28]. Current UK guidelines recommend either patient-controlled epidural analgesia (PCEA) or intermittent bolus as delivery methods of labour epidural analgesia [14]. Programmed intermittent epidural bolus (PIEB) may confer benefits compared to continuous epidural infusion and has been demonstrated as the ideal modality during labour analgesia when used in combination with PCEA [19,29]. Only a quarter of hospitals use PIEB as part of their labour analgesia technique, and a quarter use continuous epidural infusions. Remifentanil PCA availability is reported by approximately half of the hospitals. This lack of remifentanil availability may be due to a preference for other systemic opioids, safety concerns for remifentanil, or the unavailability of appropriately trained staff [30-32].

There may have been a change in clinical practices as a result of recent national shortages of diamorphine resulting in greater fentanyl or morphine use, which were more readily available at the time of data collection [33,34]. Phenylephrine infusions are used to a greater extent than previously reported, in line with consensus guidelines [35]. However, a small number of sites report the use of other non-alpha antagonist medications. There are variations in other clinical practices. Active warming (forced air warming or warmed fluid) for elective caesarean delivery decreases perioperative temperature reduction and lowers the incidence of both hypothermia and shivering [36]. However, one-quarter of sites do not routinely use such measures to prevent hypothermia. Protocolised postoperative care for caesarean delivery has been highlighted in guidelines for enhanced recovery, including the use of multimodal analgesia and antiemetics [15,22]. Our survey found that there may be scope to improve adherence to these guidelines, particularly for caesarean delivery under general anaesthesia.

There are key indicators of quality that can be used for benchmarking in obstetric anaesthesia [37]. Quality indicators are measures that reflect care or processes and are linked to improved outcomes [38]. There are recommendations for the measurement of processes and outcomes in peripartum care, which can be used for internal quality improvement, in particular, data related to interventions and complications [12,16]. We have shown that outcome measurement in obstetric anaesthesia is variable across the UK and complete data on the incidence, success, or complication rates of anaesthesia interventions is sparse. Most sites indicated that they do not record immediate outcomes such as the achievement of adequate analgesia 45 minutes after placement of an epidural [37]. The follow-up process following anaesthetic intervention occurs with greater consistency; however, this is not uniform in the way it was conducted. Furthermore, complications and adverse events such as PDPH or conversion to general anaesthesia are not recorded by all sites. Therefore, this can hinder efforts to monitor the performance within and between obstetric units and subsequent quality improvement initiatives. This may represent a further area of focus for improvement and implementation across the UK.

The survey was completed by local investigators with the aid of clinical leads to provide a snapshot of practice; the answers to survey questions may not have been known and may have been reported approximately or incompletely, which may have resulted in inaccuracy. The measures reported at a hospital site level may not reflect the care that patients actually received, and the survey did not elucidate the adherence to guidelines and protocols present at sites. This survey was conducted between surges of COVID-19, where we have previously shown there were changes in the provision of obstetric anaesthesia services [39].

This study has several limitations. We invited all obstetric units to participate in the ObsQoR study; not all did. The survey collected data from 69% of the 156 obstetric units in the UK and 93% of those units with over 6,000 deliveries per annum. The participating hospital sites in the ObsQoR study were geographically diverse and a representative sample of overall anaesthetic peripartum practices, although there was an under-representation of the smaller sites. Our response rate is favourable in the number of hospitals participating and the survey's completeness to previous studies on staffing, variation in clinical practice, complications, and outcomes in the UK [9]. We have shown statistical significance for several staffing, service provision, and facility outcomes identified between hospitals with different annual delivery numbers; the clinical significance of these findings remains unclear as the work intensity or “busyness” of an anaesthetic obstetric service (number and type of anaesthetic interventions, operating theatre procedures, critically ill patients, and pre-or postoperative anaesthetic evaluations) were not accounted for in these analyses. Whilst we have highlighted variation in a number of important aspects of obstetric anaesthetic practice within the UK, it remains unclear as to the patient-level impact on quality of care, particularly in relation to the number of deliveries at each site. In addition, we have not delineated against the strength of the recommendations reported in the guidelines for each question asked beyond those standards considered mandatory.

In summary, we identified there is variability in staffing, facilities, processes, and indicators of quality relating to obstetric anaesthesia. This may suggest scope to improve adherence to best practice guidelines and the use of implementation frameworks to prevent disparity in obstetric anaesthetic care. Further studies are needed to evaluate the relationship between benchmarking of quality indicators, the quality of postpartum recovery, and maternal outcomes.

## Conclusions

These results demonstrate variation in the provision of staffing, facilities, clinical practices, and outcome measurements across the UK. To deliver safe and equitable care across the UK, there needs to be standardisation of anaesthetic peripartum care based on national recommendations and the benchmarking and measurement of appropriate markers of quality.

## Figures and Tables

**Figure 3:**
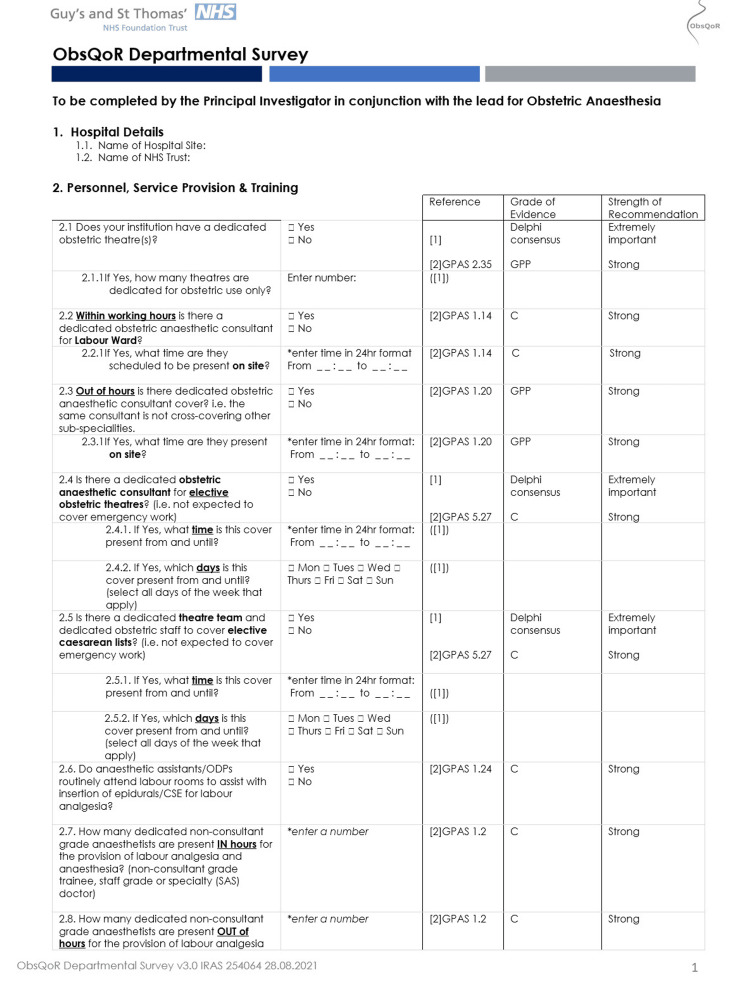
Supporting information (Page 1)

**Figure 4:**
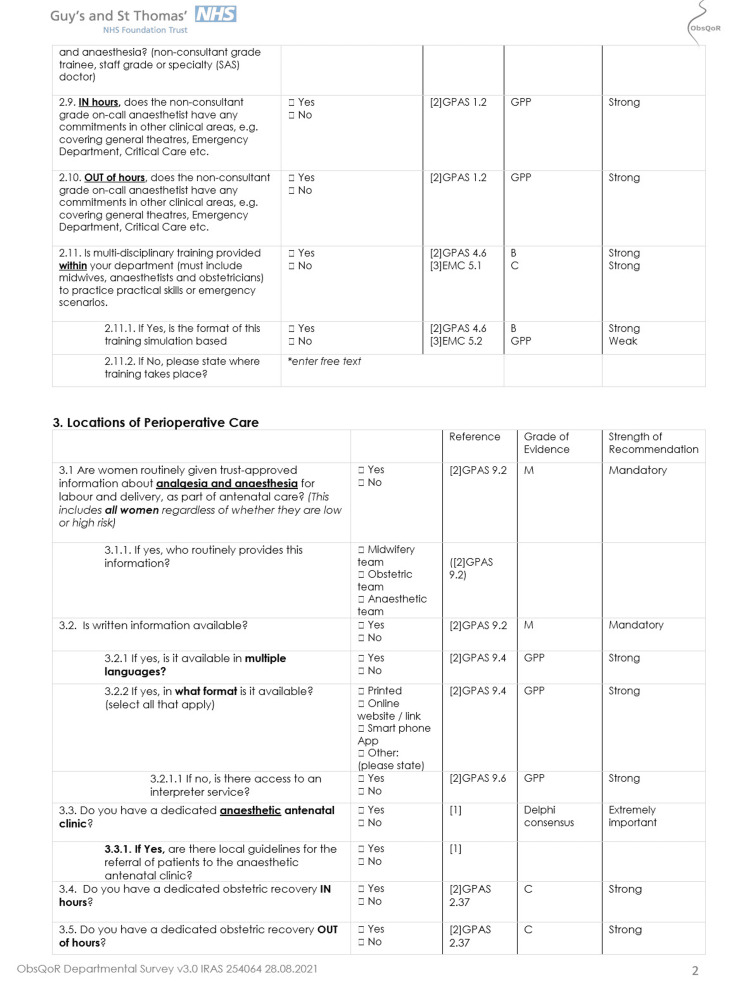
Supporting information (Page 2)

**Figure 5:**
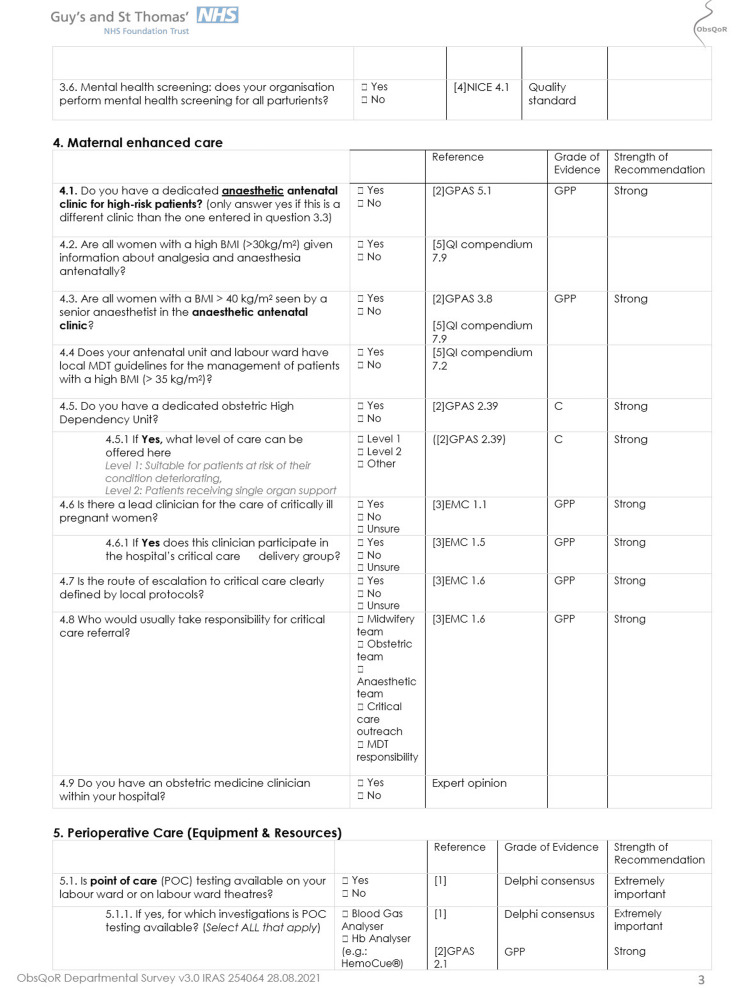
Supporting information (Page 3)

**Figure 6:**
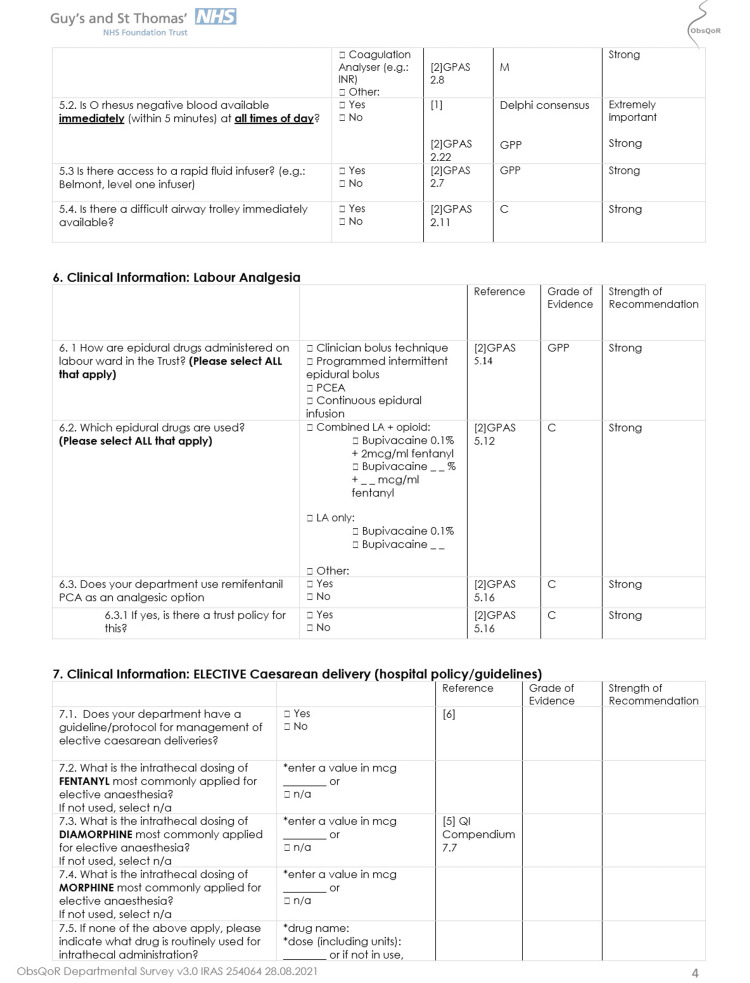
Supporting information (Page 4)

**Figure 7:**
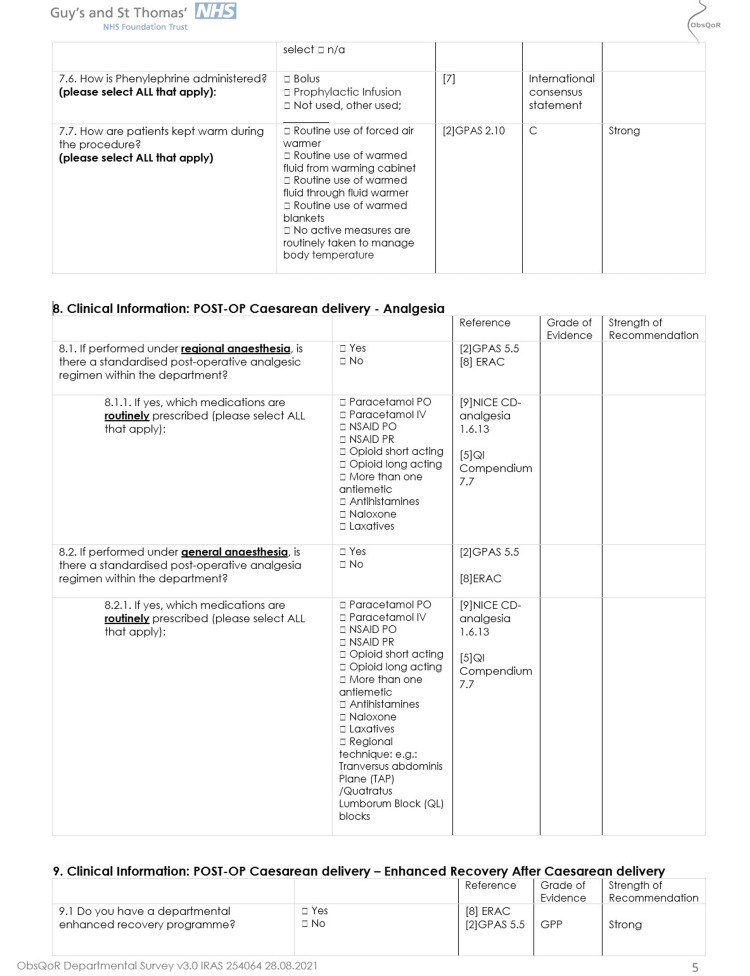
Supporting information (Page 5)

**Figure 8:**
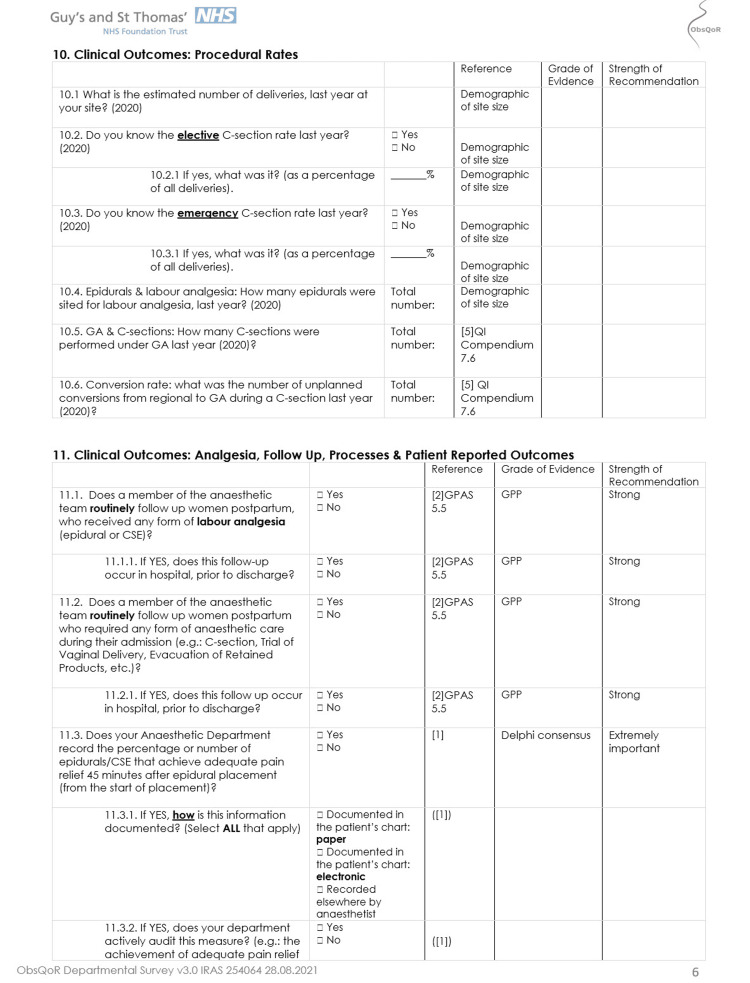
Supporting information (Page 6)

**Figure 9:**
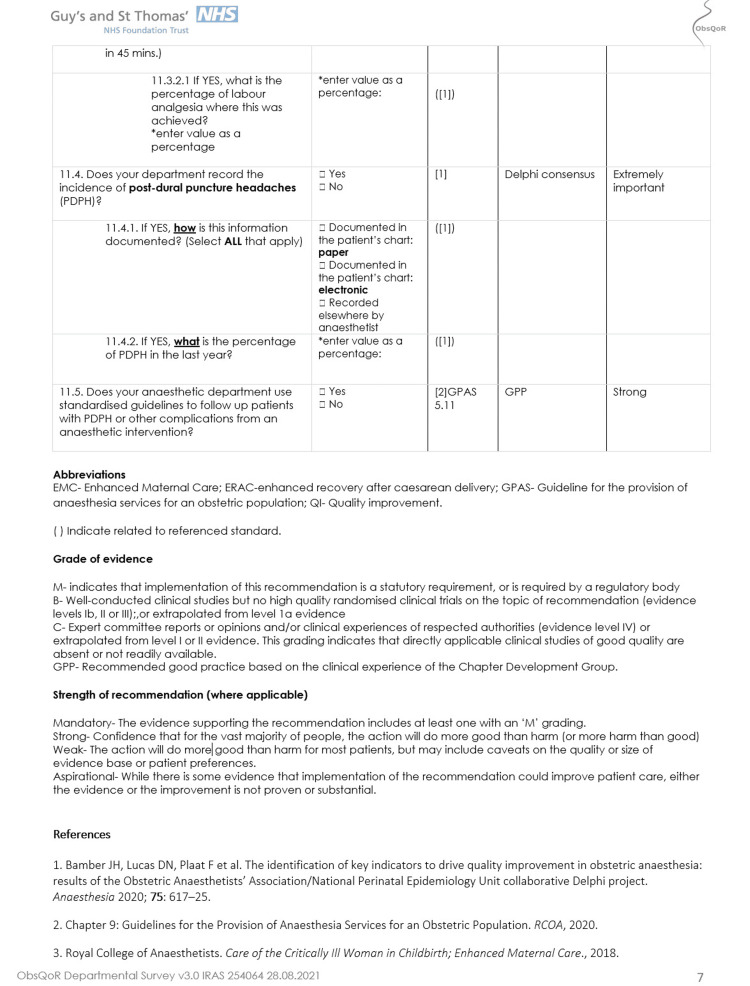
Supporting information (Page 7)

**Figure 10:**
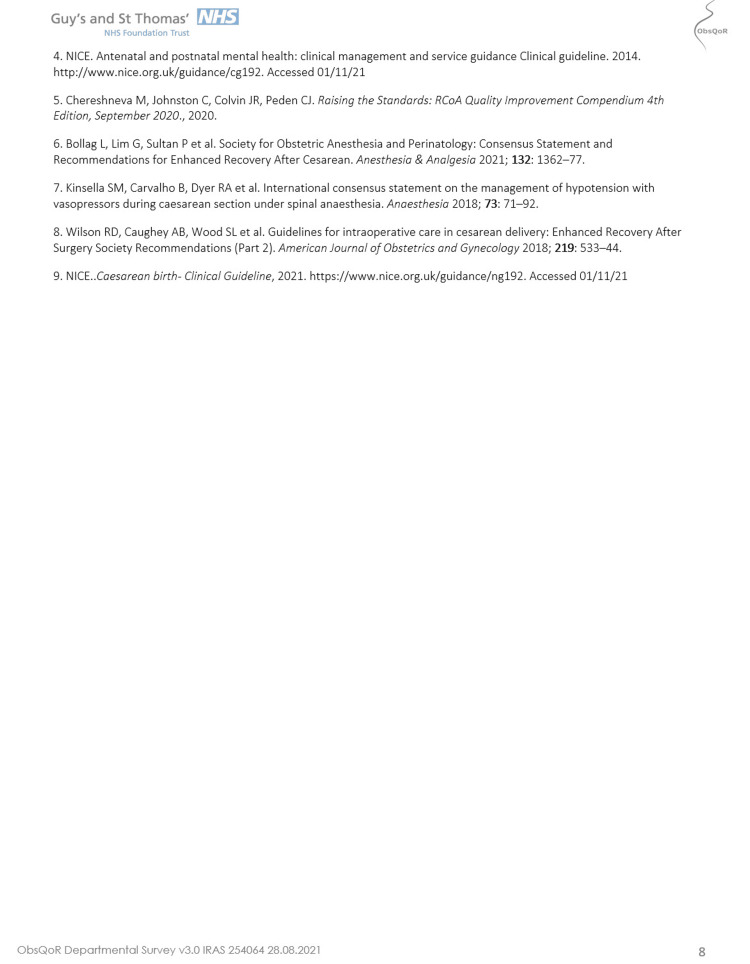
Supporting information (Page 8)

## Appendices

Appendix 1 
